# Spatial correlation reverses the compound effect of multiple stressors on rocky shore biofilm

**DOI:** 10.1002/ece3.9418

**Published:** 2022-10-27

**Authors:** Luca Rindi, Jianyu He, Lisandro Benedetti‐Cecchi

**Affiliations:** ^1^ Department of Biology University of Pisa, CoNISMa Pisa Italy

**Keywords:** covariance, environmental fluctuations, extreme events, heterogeneity, Jensen inequality, multiple stressors, variability

## Abstract

Understanding how multifactorial fluctuating environments affect species and communities remains one of the major challenges in ecology. The spatial configuration of the environment is known to generate complex patterns of correlation among multiple stressors. However, to what extent the spatial correlation between simultaneously fluctuating variables affects ecological assemblages in real‐world conditions remains poorly understood. Here, we use field experiments and simulations to assess the influence of spatial correlation of two relevant climate variables – warming and sediment deposition following heavy precipitation – on the biomass and photosynthetic activity of rocky intertidal biofilm. First, we used a response‐surface design experiment to establish the relation between biofilm, warming, and sediment deposition in the field. Second, we used the response surface to generate predictions of biofilm performance under different scenarios of warming and sediment correlation. Finally, we tested the predicted outcomes by manipulating the degree of correlation between the two climate variables in a second field experiment. Simulations stemming from the experimentally derived response surface showed how the degree and direction (positive or negative) of spatial correlation between warming and sediment deposition ultimately determined the nonlinear response of biofilm biomass (but not photosynthetic activity) to fluctuating levels of the two climate variables. Experimental results corroborated these predictions, probing the buffering effect of negative spatial correlation against extreme levels of warming and sediment deposition. Together, these results indicate that consideration of nonlinear response functions and local‐scale patterns of correlation between climate drivers can improve our understanding and ability to predict ecological responses to multiple processes in heterogeneous environments.

## Graphical abstract

Understanding how multifactorial fluctuating environments affect species and communities remains one of the major challenges in ecology. Here, we use field experiments and simulations to assess the influence of spatial covariation of two essential climate variables ‐ warming and sediment deposition following heavy precipitation ‐ on the biomass and photosynthetic activity of rocky intertidal biofilm. Our study indicate that consideration of nonlinear response functions and local‐scale patterns of covariation between climate drivers can improve our understanding and ability to predict ecological responses to multiple processes in heterogeneous environments.

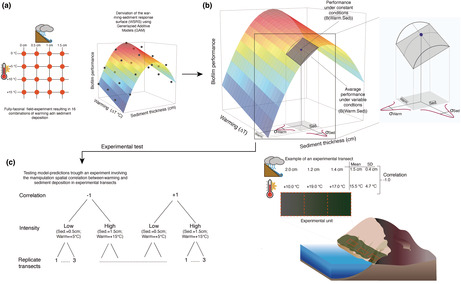

## 1 INTRODUCTION

Ecosystems face multiple interacting natural and human‐driven disturbances whose occurrence, magnitude, and impact vary in space and time (Gunderson et al., 2016; Jentsch et al., 2009; Wernberg et al., 2013). Predicting the ecological impacts of environmental fluctuations is essential as anthropogenic climate change alters the frequency and intensity of climate extremes (Drijfhout et al., 2015; Kirtman et al., 2013). Most notably, climate variability is already impacting on gross primary production at continental scales, and it is altering patterns of biodiversity at local scales (Ciais et al., 2013; Dornelas et al., 2014; Franks et al., 2007). In the last two decades, laboratory and field experiments have documented strong ecological effects of environmental fluctuations on species and assemblages through changes in frequency, variance, and autocorrelation of disturbance, resource supply, or other variables (Benedetti‐Cecchi, 2003; Benedetti‐Cecchi et al., 2006; Bernhardt et al., 2018; Bertocci et al., 2005; Crain et al., 2008; Gunderson et al., 2016; Lawson et al., 2015). One general mechanism explaining these results is the prevalence of nonlinear response functions relating ecological and environmental variables. Nonlinear responses, where a change in the input can generate a disproportionate change in the output, are ubiquitous in nature (Denny, 2017; Zhang et al., 2015). If $f\left(x\right)$ is a nonlinear function of an environmental variable x, nonlinearity causes a mismatch between the expected response under average conditions, $f(\bar{x}$), and the integrated response under variable condition, $\bar{f\left(x\right)}$, such that $\bar{f\left(x\right)}\neq f\left(\bar{x}\right)$. This mathematical property of nonlinear functions is known as Jensen's inequality or nonlinear averaging (Jensen 1906). The sign of the inequality is positive (negative) for accelerating (decelerating) response functions, whereas the magnitude of change depends on the degree of nonlinearity and the amount of variability in × (Chesson, 2012; Ruel & Ayres, 1999). Ecologists have successfully used Jensen's inequality to model the performance of producers and consumers under stressful and variable environmental scenarios (Benedetti‐Cecchi, 2005; Benedetti‐Cecchi et al., 2012; Bernhardt et al., 2018; Denny, 2017; Koussoroplis & Wacker, 2016; Ruel & Ayres, 1999; Vasseur et al., 2014).

The great emphasis on large temporal and spatial scales of variation in environmental conditions has overshadowed the importance of local‐scale environmental variability (Helmuth et al., 2006; Sears et al., 2011). Yet, predictions and ecological outcomes at local scales can be dramatically different from those generated by global climate models (Nadeau et al., 2017). Landscape configuration and heterogeneity have been shown to play an essential role in modulating the impact of large‐scale climate forcing on both plants and animals (Dong et al., 2017; Lehtilä et al., 2020; Ohler et al., 2020; Sunday et al., 2014). Spatial heterogeneity in the thermal environment may create local refugia, allowing species to survive during unusually harsh conditions (e.g., during heatwaves). For example, local‐scale microclimatic conditions in plant communities can determine long‐term resilience in rear edge forests again heatwaves and drought pressures (Carnicer et al., 2021). In addition, spatial heterogeneity may also ensure the persistence of thermally sensitive species (Angilletta, 2009). However, the extent to which spatially variable thermal regimes determine the performance of organisms in natural environments remains largely understudied (Dowd et al., 2015).

Despite the widely recognized importance of environmental variability in influencing organisms' performance, few studies have explored the consequences of Jensen's inequality in a multifactorial context, incorporating the effect of correlation between variables (Koussoroplis & Wacker, 2016; Koussoroplis et al., 2019; Pincebourde et al., 2012). The simultaneous effect of multiple factors may produce nonlinearities that would remain undetected in a unifactorial scenario (Koussoroplis et al., 2017). These nonlinearities may interact with environmental variance and the degree and direction of the correlation among environmental factors, leading to a deviation between organism's performance under constant and variable conditions. Direct evidence of the role of correlation in modulating organisms' performance stems from recent laboratory and mesocosm experiments (Koussoroplis & Wacker, 2016; Koussoroplis et al., 2019; Pincebourde et al., 2012). For instance, Koussoroplis and Wacker (2016) have shown how correlation between temperature and resource supply influenced the life‐history traits of the water flea Daphnia magna. To what extent the variance and correlation between simultaneously fluctuating variables affect ecological assemblages under field conditions remains largely untested.

Rocky intertidal habitats have been extensively used as model systems to test cornerstone hypotheses in ecology and to unravel the influence of multiple interacting processes (Hawkins et al., 2020). Plants and animals living on rocky shores are exposed to a mosaic of environmental conditions where key variables such as temperature and wave action often covary at experimentally tractable spatial scales (Helmuth et al., 2006; Lima & Wethey, 2012; Hawkins et al., 2020). The small size and short life span of many organisms living on rocky shores further facilitate the analysis of multiple stressors and their correlation in space or time. For example, recent studies have used epilithic microphytobenthos (hereafter, biofilm) to show how the temporal clustering of extreme events of warming and sediment deposition can promote legacy effects and drive populations to collapse (Dal Bello et al., 2017, 2019). These and other studies have documented strong negative effects of warming and sediment accretion following heavy rains on rocky intertidal organisms (Harley, 2003; Kordas et al., 2015; Vaselli et al., 2008). Substratum complexity, generated by emergent surfaces mixed with heterogenous areas with depressions, cracks, and crevices in the rock, can result in different patterns of spatial correlation between aerial temperature and sediment. For example, positive correlation may occur on emergent rocks on sunny days and calm sea, whereas cracks and crevices where sediment is more persistent during rough sea conditions may introduce negative correlation.

We used a combination of experimental and simulation approaches to evaluate how spatial correlation between two important climate variables, warming and sediment accretion following run‐off, modulated the performance of rocky intertidal biofilm. As a first step, we used a response surface design involving 16 combinations of warming and sediment deposition to derive a response surface (hereafter RS) relating the biomass and photosynthetic activity of biofilm to the two variables in the field. Then, we used the empirically derived RS to simulate the performance of biofilm under different correlation scenarios of warming and sediment deposition. We expected the performance of biofilm to differ between constant and variable conditions owing to nonlinearities in the RS. Finally, we tested the predictions originating from our simulations in a second field experiment in which we manipulated the spatial correlation (positive or negative) and intensity of warming and sediment deposition in a factorial experiment. Our results provide the first empirical evidence of how spatial correlation between interacting stressors and nonlinear effects drives small‐scale ecological responses of primary producers in real‐world conditions and indicate that local‐scale patterns between climate variables may play a crucial role in predicting ecological responses to multiple processes in heterogeneous environments.

## 2 METHODS

### 2.1 Study site

The study was done along the coast of Calafuria (Livorno, 43° 30′ N, 10°19′ E) between March and December of 2017. The coast is composed of gently sloping sandstone platforms with high‐shore levels (0.3–0.5 m above mean low‐level water) characterized by populations of barnacles interspersed among areas of seemingly bare rock, where biofilm develops. Biofilm at Calafuria is composed mainly of cyanobacteria of the genus *Rivularia*, contributing up to 50% of the bacterial assemblage (Maggi et al., 2017) (Figure S1a). As shown in a previous study, warming and sediment deposition following run‐off are important drivers of biofilm biomass in this system (Dal Bello et al., 2017). Grazing by the littorinid snail *Melarhaphe neritoides* (L.) can also affect the abundance and distribution of the biofilm, mostly in late fall and winter (Dal Bello et al., 2017).

### 2.2 Experiment 1: Derivation of the response surface

**FIGURE 1:**
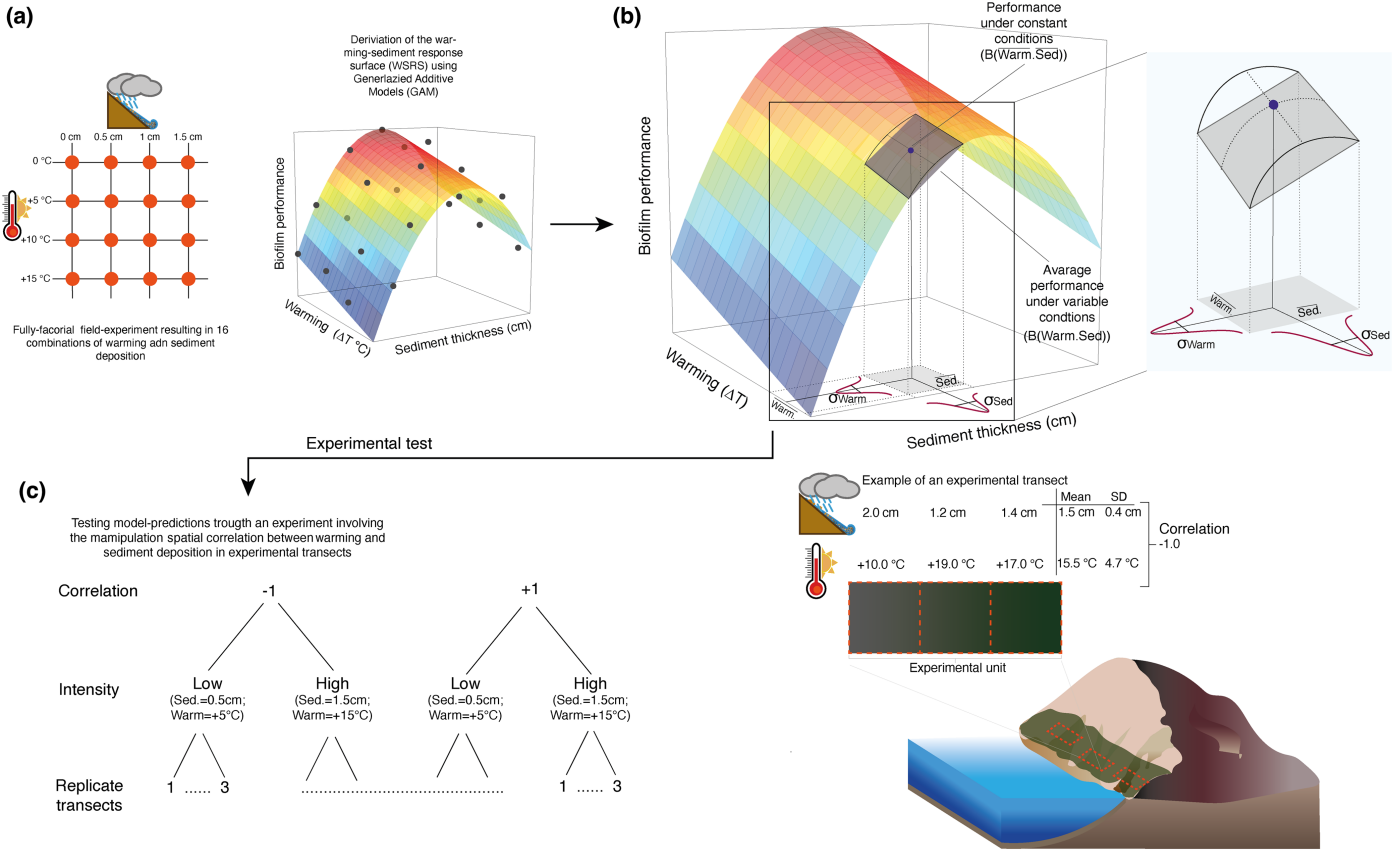
Flow chart illustrating the main steps of the study. (a) To derive a response surface, we performed a full factorial experiment crossing four warming intensities (+0°C, +5°C, +10°C, and +15°C above ambient air temperature, corresponding to 24.8°C, 30.4°C, and 40.0°C absolute temperatures, respectively) with four levels of sediment deposition (0 cm, +0.5 cm, +1.0 cm, and + 1.5 cm thick layers of sediment deployed over the plots). (b) Hypothetical warming and sediment response surface. The response surface was used to generate predictions of biofilm performance under variable ($\bar{B\left(\text{Warm}.,\mathrm{Sed}.\right)}$) and constant ($B\left(\bar{\text{Warm}.},\bar{\mathrm{Sed}.}\right)$) conditions for different scenarios of spatial correlation between warming and sediment deposition. The inset shows the effect of nonlinear averaging, – i.e., the deviation of biofilm performance due to the nonlinearity characterizing the warming‐sediment RS – for a specific combination of warming and sediment deposition. Warming and sediment deposition are expected to follow a normal bivariate distribution. (c) We performed a field experiment to test the predicted outcomes of the simulations by manipulating the intensity and spatial correlation of warming and sediment deposition along experimental transects. (d) Transects consisted of three contiguous quadrats of 40 × 40 cm. Spatial correlation between warming and sediment deposition was generated by varying the levels of the two variables along the quadrats in a transect. The panel shows a high intensity treatment with an average level of warming of +15.5°C above ambient temperature, an average layer of sediment of 1.5 cm, and a level of spatial correlation between the two variables of −1.

We used a response‐surface experimental design involving 16 combinations of warming and sediment deposition to build a warming‐sediment response surface (RS) (Figure 1a). The experiment was conducted between May and August 2017. In May 2017, 48 plots consisting of patches of rock 40 × 40 cm fully covered by biofilm were marked at their corners with rawl plugs inserted into the rock for future relocation. Three replicated plots were randomly allocated to each combination of four levels of warming crossed with four levels of sediment deposition. Temperature and sediment were manipulated following methodologies developed in previous studies (Dal Bello et al., 2017). The warming factor included a control (ambient temperature) and three levels of elevated temperatures (+5°C, +10°C, and +15°C above ambient levels). Plots were heated with aluminium chambers equipped with stoves (Figure S1), with warming levels chosen to reflect a wide range of temperatures experienced by the biofilm at the study site, from common to rare. We characterized individual warming levels as the averaged return time of positive thermal anomalies using a 66‐year time series of temperature measurements (Appendix S1, Figure S2). Positive temperature anomalies of +5°C are common during the central hours of the day and have a return time of less than 1 year, while anomalies of +10°C have a return time of about 2 years. Anomalies of +15°C corresponded to extreme conditions with a return time of about 85 years. The warming treatment consisted of keeping the difference between the chamber and the ambient air temperature as close as possible to the designated treatment level for 2 h (Figures S1c, S3a,b). The aerial temperature was constantly measured with iButton loggers during the 2 h of warming inside and outside the heating chambers. Three additional plots were established as control for artifacts (CA) to assess the potential effect of shading on biofilm during the heating sessions. CA plots were shaded with cardboard chambers, but they were not warmed.

Sediment deposition included a control (no sediment added) and three levels of sediment accretion (+0.5 cm, +1.0 cm, and +1.5 cm thick layers of sediment deployed over the plots), to mimic the effects of runoff following heavy rainfall events (Figure S1d). Sediment layers about 0.5–1 cm thick originated naturally on flat rocks after intense storms (>70 mm within the previous 24 h) and could persist for about 2/3 days before being washed away by waves (Figure S1b) (Dal Bello et al., 2017). In some areas, depressions on the rock favored sediment accretion with the formation of mats up to 15 mm thick. We mimicked these events by adding to each designated plot a layer of sediment collected in the surrounding area and diluted in freshwater. Sediment thickness was measured with a caliper and adjusted accordingly to the nominal value of the sediment deposition treatment assigned to each plot (Figure S1c). Warming was performed before sediment deposition, but the order in which the two stressors are imparted has no effects on the biofilm (Dal Bello et al., 2017). Experimental units were monitored in the 2–3 days following treatment application to assess whether sediment thickness matched nominal treatment levels and to adjust when necessary. Experimental treatments were applied only once as a single pulse; due to the impossibility of treating all the 51 plots (48 used to derive the response surface and the three CA plots) on the same day, sets of 5–6 randomly chosen plots were treated in each of 6 days within a month.

Biofilm biomass and photosynthetic activity were evaluated after 7, 14, and 21 days since the start of the experiment. Biomass was determined by means of an image‐based remote sensing technique that uses chlorophyll *a* concentration as a proxy (μg chl *a* cm^−2^). Chlorophyll *a* was estimated from a ratio of reflectance at near‐infrared (NIR) and red bands (Ratio Vegetational Index – RVI) by means of a IR‐sensible camera (ADC), following the method proposed by Murphy and colleagues (Murphy et al., 2009). NIR/red ratios are related to chlorophyll *a* by a linear relationship, calculated on the basis of laboratory chlorophyll *a* extraction from Calafuria sandstone cores (Dal Bello et al., 2015). Each photo was then handled with a java‐routine in ImageJ software to haphazardly select six subplots and provide a mean value of biofilm biomass for each plot (Schneider et al., 2012).

The physiological status of the photosynthetic apparatus of biofilm was assessed through a portable underwater pulse‐amplitude‐modulated (PAM) fluorometer (Diving‐PAM, Walz). Maximum photochemical efficiency after 5′ of dark adaptation (henceforth dark yield) and effective quantum yield of photosystem II in actinic light (henceforth light yield) were used as a proxy of photosynthetic efficiency and stress, respectively. Within each experimental plot, three and six measurements were haphazardly taken for light and dark yield, respectively. Sampling had an average duration of about 2 h and started around 2.5 h after sunrise and ended at midday. Dark yield measurements were taken after 5 min of dark adaptation, while light yield was measured under natural light condition.

We used a generalized additive model (GAM) to derive the RS from the experimental data.

We obtained a single RS for each response variable (Chl *a*, dark and light yield) by taking averages over the three sampling dates. Data were modeled as a function of three smoothers of nominal levels of warming (*W*) and sediment deposition (*S*) and their interactions (*W* × *S*) (with identity link and Gaussian error distribution):

(1) $$Y_{\mathrm{ijk}}\sim \beta_{0}+s_{1}\left(W_{i}\right)+s_{2}\left(S_{j}\right)+\mathrm{te}\left(W\times S_{\mathrm{ij}}\right)+\varepsilon_{\mathrm{ijk}}$$

where the *Y*_*ijk*_ is the value of the response variable (Chl *a*, dark and light yield) in replicate *k*, sediment level *j*, and warming level *i*, *ß*_0_ is the intercept, *s*_1_ and *s*_2_ are thin plate regression splines describing the individual effect of warming and sediment, *te* is tensor product smooth term modeling the interaction between warming and sediment, and ε is the Gaussian error term. Smoother terms were selected through a generalized cross‐validation procedure. The number of knots was set to 4, which corresponded to the number of levels of predictors variables. Model assumptions were assessed visually using plots of residuals vs. fitted values, box plots of residuals vs. experimental conditions, and QQ plots of standardized residuals vs. normal quantiles (Faraway, 2016). GAM fitting was performed using the function gam of package mgcv in R 3.5.1 (Wood et al., 2016).

Potential artifacts due to shading effects during warming sessions were assessed through a *t*‐test contrasting control and CA plots.

### 2.3 Simulating from the response surface

The RS experiment identified a significant nonlinear response of biofilm biomass to warming and sediment deposition (in interaction), but not for photosynthetic activity (see RESULTS – Experiment 1: Derivation of the response surface). Thus, we used the RS model derived for biomass to simulate the response of biofilm to changes in warming and sediment deposition under constant and variable scenarios and for different patterns of correlation between the two stressors.

These simulations allowed us to explore the interactive effects between nonlinearities and environmental variance on biofilm performance and the modulating effect of spatial correlation. We started our simulations by generating values of biofilm biomass under constant ($Y_{\text{const},\mathrm{ij}})$ and variable ($Y_{\mathrm{var},\mathrm{ij}}$) conditions, where *i* indicated values of temperature (*T*_*i*_, with *i* varying between 0 and 20°C in steps of 0.5) and *j* the levels of sediment deposition (*S*_*j*_, with *j* varying between 0.5 and 1.5 cm in steps of 0.5), resulting in a prediction grid of 164 values. Biomass values under constant conditions were simulated by simply feeding the RS model with the warming and sediment deposition values. To simulate variable conditions, for each point in the prediction grid, we generated 1000 values of temperature and sediment deposition by sampling a bivariate normal distribution $\left(X\sim N\left(\mu ,\sum \right)\right)$ with mean $\mu =\left(\begin{array}{c} \mu_{T}\\ \mu_{S} \end{array}\right)$ and covariance $\sum =\left(\begin{array}{cc} \sigma_{T}^{2} & \rho \sigma_{T}\sigma_{S}\\ \rho \sigma_{T}\sigma_{S} & \sigma_{S}^{2} \end{array}\right)$, where $\mu_{T}$ and $\mu_{S}$ corresponded to the chosen prediction values, ρ defined the strength of correlation between predictors, and $\sigma_{T}$ and $\sigma_{S}$ were the standard deviations of temperature and sediment thickness estimated from field measurements during the experiment ($\sigma_{T}$ = 4.54°C and $\sigma_{S}$ = 0.41 cm) (Figure S4). In particular, $\sigma_{T}\;$was calculated from temperatures in control plots, while $\sigma_{S}$ was calculated from data of the thickness of sediment deposits taken after heavy rainfall events. Thus, the two standard deviations $\sigma_{T}$ and $\sigma_{S}$ quantified the natural levels of spatial variability of warming and sediment deposition at the study site. We used the mean over 1000 simulations to obtain the predicted value of biofilm biomass for each combination of temperature and sediment deposition from the RS model. This procedure was repeated for different values of correlation (ρ) ranging from −1 to 1, with increments of 0.2.

In addition to recording changes in biofilm biomass in the various scenarios, we used the simulated values to compute the total variance effect (TVE), a quantity that summarizes the effect of variance and correlation between stressors (Koussoroplis & Wacker, 2016). The TVE (in percentage) for a given combination of warming *i* and sediment deposition *j* was computed as:

(2) $$\mathrm{TVE}\left(\%\right)=\frac{Y_{\mathrm{var},\mathrm{ij}}-Y_{\text{const},\mathrm{ij}}}{Y_{\text{const},\mathrm{ij}}}\times 100\;$$

This metric expressed the standardized percentage difference of biomass under variable conditions compared with constant ones. Therefore, negative values of TVE corresponded to lower values of biomass under variable than constant conditions, resulting in a negative effect of variance on biomass. The opposite applied to positive values of TVE.

### 2.4 Experiment 2: Testing predictions

Simulations generated quantitative predictions on the nonlinear response of biofilm biomass to changes in variance and correlation between warming and sediment deposition. We tested some of these predictions in a second experiment in which we manipulated the intensity and spatial correlation between stressors. In August 2019, we marked 12 transects each consisting of three contiguous quadrats (40 × 40 cm) in areas originally covered by biofilm (Figure S1d). The experiment had a factorial design with two levels of correlation (+1 and −1) crossed with two levels of intensity (low: +5°C of warming and 0.5 cm of sediment deposition; high: +15°C of warming and 1.5 cm of sediment deposition) and three replicate transects in each treatment combination. Each transect constituted a single experimental unit in which spatial correlation was manipulated by exposing each of the three quadrats to a specific combination of warming and sediment deposition. For example, in the positive correlation treatment, quadrats exposed to high (low) temperatures also received high (low) levels of sediment deposition. In contrast, in the negative correlation treatment, quadrats exposed to low (high) warming also received high (low) levels of sediment deposition (Figure 1d). Initial values of temperature and sediment for the three quadrats in each transect with designated levels of correlation (+1 and −1) were generated by sampling a bivariate normal distribution (Figures 1d, S1c,d). Due to the small sample size involved (the three quadrats in a transect), this procedure often resulted in levels of spatial correlation that were lower than the nominal level. In these instances, the values of the two variables were adjusted arbitrarily to obtain the desired level of correlation. As in the first experiment, three additional transects were used as controls for artifacts (CA) to assess the potential effect of shading on biofilm during the heating sessions.

To compute the TVE, each of the four combinations of intensity and correlation of stressors was matched with an independent set of three replicate transects (12 in total: three replicates × two levels of correlation × two levels of intensity) with zero correlation between warming and sediment deposition. These additional transects provided the constant condition at the denominator of Equation 2 and were obtained by imparting the same levels of warming and sediment deposition across the three quadrats in each transect. We recognize that although these treatments had zero nominal covariance, realized correlations could differ from zero owing to small variation in treatments levels across quadrats in a transect. Nevertheless, since Equation 2 uses the mean values of biofilm biomass across treatments, it is the realized mean correlation that must be close to zero. We verified this expectation by recording the amount of warming and sediment deposition imparted to all quadrats during the experiment and computing the corresponding correlation values (Figure S4).

Chl *a* was used as a surrogate for biofilm biomass and was measured 10 and 18 days after the beginning of the experiment as described above (Section 3.1). Treatment effects were evaluated on time‐averaged Chl *a* and TVE values in each quadrat using Linear Mixed Effect Models (LMEM) (Bates et al., 2015). In the model, Correlation (with two levels: −1 and +1), Intensity (with two levels: Low and High), and their interactions (Correlation × Intensity) were included as fixed effects, while transects were included as a random effect to account for the lack of spatial independence among biomass values in different quadrats. Post‐hoc contrasts between treatments were performed using the “emmeans” package in R (Lenth et al., 2018). We evaluated model assumptions using standard graphical procedures. 95% confidence intervals (CIs) were computed for each experimental condition using non‐parametric bootstrapping and were used to assess the convergence of experimental and predicted values of biomass and TVE. The bootstrap procedure involved resampling with replacement each experimental condition 1000 times. The 95% CIs were then calculated as 2.5th and 97.5th percentile of the distribution of bootstrapped values.

To assess the effect of error propagation stemming from the uncertainty associated with the RS, we calculated the 95% CIs of predicted values of biomass and TVE using a bootstrapping technique. We followed a three‐step process: (1) the biomass values obtained in the first experiment for each combination of warming and sediment deposition were sampled with replacement to generate a bootstrapped dataset; (2) the GAM model (Equation 1) was then fed with the bootstrapped data to generate biomass and TVE values for each of the four treatment combinations examined in the second experiment (see Section 2.3); (3) 95% CIs were finally computed as 2.5th and 97.5th of the vector of bootstrapped estimates, obtained by repeating the steps 1 and 2 above 1000 times.

Data and R‐script used in this study are available from Figshare (DOI: 10.6084/m9.figshare.14447871).

## 3 RESULTS

### 3.1 Experiment 1: Derivation of the response surface

**FIGURE 2:**
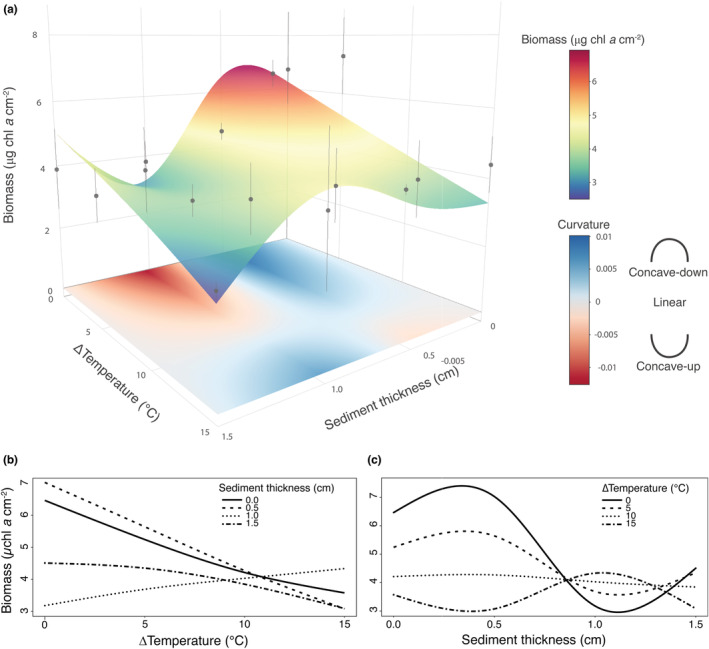
Biofilm biomass as a function of warming and sediment deposition. (a) Response surface (RS) relating biofilm biomass to warming and sediment deposition. Blue regions of the curve indicate positive values of the curvature (approximated by its second derivative) and reflect a concave‐down region of the RS, while red regions indicate a locally concave‐up curvature. Data shown are mean values of biofilm biomass (*n* = 3) estimated as μg chl *a* cm^−2^ for each combination of warming and sediment deposition. Cross sections of the response surface are shown across levels of warming (b) and sediment deposition (c).

Warming and sediment deposition interactively affected biofilm biomass (GAM: *Wald*_*Warm×Sed*_ = 1.09, *p* < .01; Figure 2a, Table S1). The RS showed four distinct regions with contrasting nonlinear responses of biofilm biomass to warming and sediment deposition. A concave‐8(0–10°C) and at intermediate sediment deposition (1 cm) and extreme warming (15°C) (Figure 2a). In contrast, a concave‐up relation was evident at low to moderate levels of warming (0–10°C), at moderate to extreme levels of sediment deposition (1–1.5 cm) and, to a less extent, under extreme warming and low sediment deposition (Figure 2a). A response surface fitted to absolute temperatures (Figure S5) provided a similar outcome, although it explained slightly less variation (delta temperatures in Figure 2a: AIC = 175.0, *R* ^2^_Adj._ = 29%; absolute temperatures in Figure S5: AIC = 178.71, *R* ^2^_Adj._ = 27.7%).

Cross sections of the RS indicated the prevalence of a declining, almost linear relations between biomass and warming for all but the intermediate levels of sediment deposition, where the relation became positive (Figure 2b). In contrast, biomass changed nonlinearly with sediment thickness for all but the intermediate level of warming (Figure 2c). The relation was concave‐down at low to moderate levels of warming and sediment deposition, becoming concave‐up at the most extreme level of sediment thickness. The opposite was observed under extreme warming (Figure 2c).

Neither dark nor light yield exhibited a significant relationship with warming and sediment deposition (Figure S6, Table S2). Shading effects or other artifacts due to the heating chambers were not detected for any of the three variables examined (Figure S7, Table S4).

### 3.2 Simulations

**FIGURE 3:**
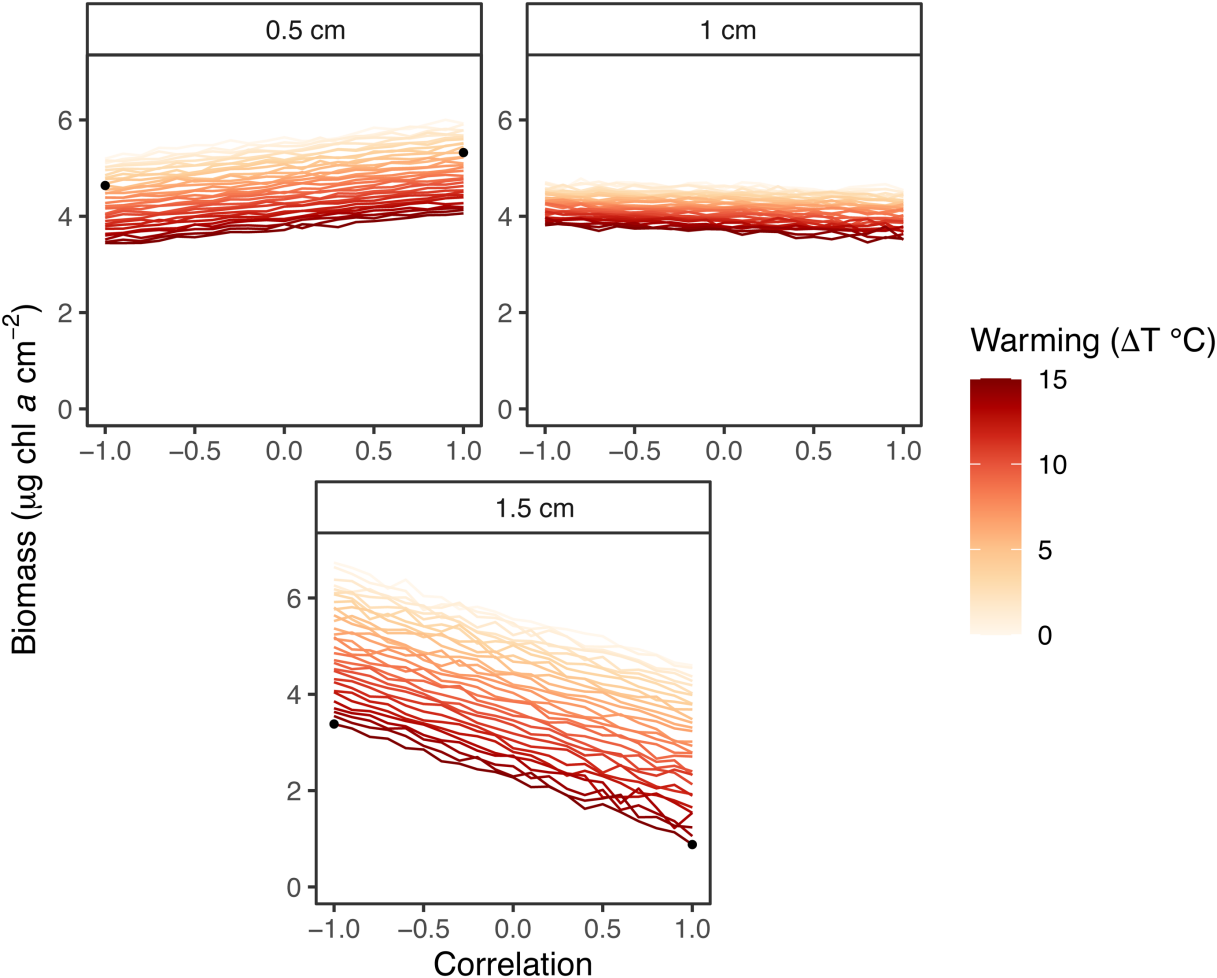
Biomass simulations for different correlation scenarios of warming and sediment deposition. Panels show the response of biofilm biomass (as μg chl *a* cm^−2^) for different levels of warming, sediment deposition, and their spatial correlation in simulations. Black dots indicate the values of the two variables and their correlation chosen as treatment levels in the subsequent experimental test of simulation predictions.

The strength and direction of correlation between warming and sediment deposition modulated the compounded effect of these stressors on biofilm biomass (Figure 3). At the lowest level of sediment deposition (0.5 cm), biofilm biomass exhibited a slight positive relationship with the degree of correlation between the two stressors, while at the intermediate level of sediment thickness (1.0 cm), biofilm showed no variation along the correlation gradient (Figure 3a,b). Under extreme sediment deposition (1.5 cm), biomass drastically decreased with increasing correlation, collapsing at high levels of warming (Figure 3c). Warming reduced biofilm biomass across all levels of sediment deposition (Figure 3a–c).

**FIGURE 4:**
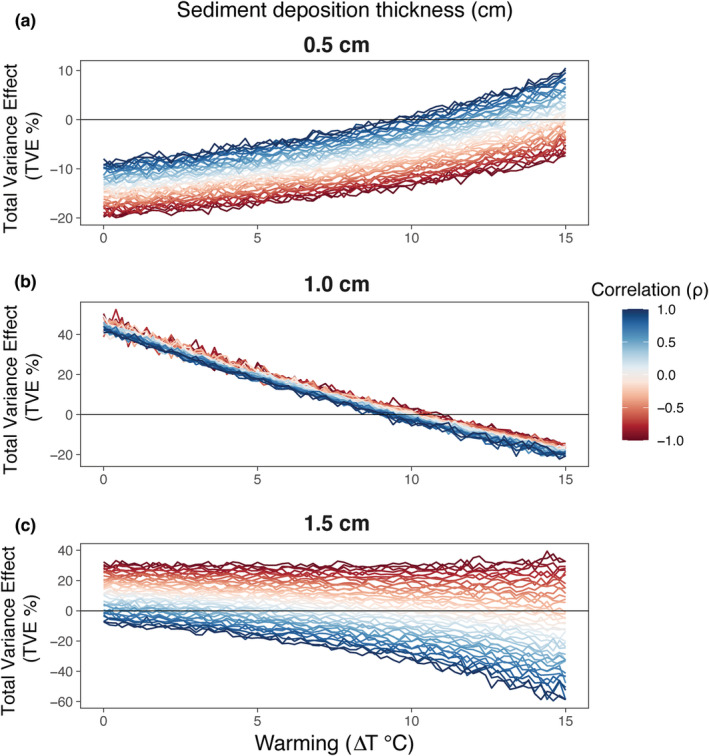
Total variance effect (TVE). Joint warming‐sediment variance and correlation effects on biofilm biomass (μg chl *a* cm^−2^) as a function of warming (∆*T*) at each of three levels of sediment deposition: 0.5 cm (a), 1.0 cm, (b) and 1.5 cm (c). The TVE quantifies the change of biofilm biomass between variable and constant conditions for a given combination of sediment and warming.

Fluctuating conditions of low to moderate warming (0–10°C) and low sediment deposition depressed biofilm biomass compared with a constant environment, whereas environmental variability became beneficial to biofilm at larger temperatures (18–20°C), determining a shift from a negative to a positive TVE (Figure 4a). This trend reversed at the intermediate level of sediment deposition, with the TVE declining consistently along the warming gradient (Figure 4b). The TVE exhibited a funnel‐shaped pattern along the warming gradient at the extreme level of sediment deposition, with negative (positive) correlation driving positive (negative) effects of variance on biofilm biomass (Figure 4c). Overall, the effect of a negative correlation between stressors on the TVE shifted from negative to positive with increasing levels of sediment deposition (compare Figure 4a with c).

### 3.3 Experiment 2: Testing predictions

**FIGURE 5:**
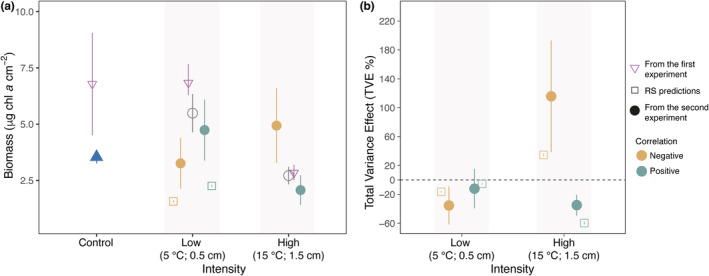
Testing predictions. Predicted vs. experimental values of biomass (μg chl *a* cm^−2^) as a function of the correlation and intensity of warming and sediment deposition. Yellow and dark‐green filled circles indicate mean biofilm biomass under negative and positive correlation, respectively (*n* = 3). Gray circles refer to mean biomass of transects exposed to constant conditions (*n* = 3). Purple reversed‐triangles indicate mean biomass of plots exposed to constant conditions derived from the first experiment (*n* = 3). Yellow and dark‐green empty squares are the expected values of biofilm biomass obtained from simulations under low (∆*T* = +5°C and sediment deposition = 0.5 cm) and high (∆*T* = +15°C and sediment deposition = 1.5 cm) intensity, respectively. Error bars are non‐parametric bootstrapped 95% confidence intervals. In particular, error bars of predictions incorporate the effect of error propagation stemming from uncertainty associated with the RS.

The impact of warming and sediment deposition on biofilm was strongly modulated by the degree of spatial correlation between these stressors (Figure 5a). The analysis identified a significant interaction between correlation and treatment intensity (LMEM for the Correlation × Intensity interaction: *t* = 2.98, *p* < .05, df = 12; Figure 5a, Table S3). Negative correlation increased significantly biofilm biomass compared with the positive correlation treatment at high intensity of warming and sediment deposition, whereas the opposite (not significant) pattern occurred when the two stressors were imparted at low intensity (Figure 5a, post‐hoc contrasts, Table S3). Observed values of biofilm biomass were close to simulated values, and differences could be considered not significant under the more intense conditions of warming and sediment deposition where treatment means were embraced in the simulated CIs (Figure 5a). In agreement with the outcomes of the simulations, negative correlation was beneficial to biofilm biomass under the most stressful conditions, whereas the opposite was observed under low intensity of warming and sediment deposition (Figure 5a).

Similarly to what observed for biofilm biomass, spatial correlation and treatment intensity interactively affected the TVE, (LMEM for the Correlation × Intensity interaction: *t* = 3.23, *p* < .01, df = 12; Figure 5b, Table S3). The TVE was significantly larger for negative compared with positive correlation at high intensity of warming and sediment deposition, whereas the opposite (not significant) pattern was observed at low treatment intensity (post‐hoc contrasts, Table S3). Similarly to what observed for biomass, the experimental results for the TVE matched the expectations originating from simulations; observed and expected values were statistically undistinguishable under positive correlation (treatment means were within the simulated CIs) (Figure 5b).

LMEMs indicated no differences between controls for artifacts and controls for the three response variables examined (Figure S8, Table S5).

## 4 DISCUSSION

Jensen's inequality has provided a phenomenological model for interpreting and predicting how environmental fluctuations and nonlinear response functions interactively affect organisms' performance (Denny & Benedetti‐Cecchi, 2012; Ruel & Ayres, 1999). Recent laboratory studies have extended Jensen's inequality to a multifactorial context, showing how correlation between multiple ecological drivers can shape the response of organisms to environmental fluctuations (Pincebourde et al., 2012, 2016; Koussoroplis & Wacker, 2016; Koussoroplis et al., 2017–2019). Consideration of multiple factors and their correlation provides a more realistic view of the performance of organisms in fluctuating environments, compared with the analysis of individual factors. Yet, empirical tests of these ideas in real‐world conditions have lagged behind theory (Chesson, 2012; Koussoroplis et al., 2017).

Combining simulations with field experiments, our study showed how the degree of spatial correlation between warming and sediment deposition modulated the impact of these stressors on rocky intertidal biofilm through the total variance effect (TVE). In principle, the direction and magnitude of the TVE for a specific combination of warming and sediment deposition should reflect the degree and direction (concave‐up or concave‐down) of the curvature of the response surface (RS) (Denny & Benedetti‐Cecchi, 2012). Jensen's inequality correctly identifies the direction of nonlinear averaging effects for univariate response functions (Benedetti‐Cecchi, 2005; Ruel & Ayres, 1999; Foray et al., 2014; Wetzel et al., 2016). In contrast, predicting from response surfaces (multiple predictors) requires consideration of the direction and degree of correlation between variables. In our analysis, outcomes consistent with Jensen's inequality were observed for regions of the RS where deviations from linearity (i.e., the curvature) were more pronounced. Negative values of the TVE corresponded to a strong positive curvature of the RS (concave‐down), such as those obtained at low and intermediate levels of sediment deposition in combination with either low or elevated warming, respectively. In contrast, positive values of the TVE were observed at intermediate levels of sediment deposition and low warming, where the RS had a strong negative curvature (concave‐up). The modulating effect of spatial correlation was not strong enough to change the sign of the TVE for these combinations of stressors (Figures 2 and 4).

The correlation effect – i.e. the contribution of spatial correlation to nonlinear averaging – emerges when two stressors act non‐additively, that is, when the effect of the concurrent change of two stressors is different from the sum of the effects of changing each stressor individually (Crain et al., 2008; Koussoroplis et al., 2017). In our simulations, the moderate correlation effect in the low‐intensity condition originated from the mild antagonistic effect of warming and sediment deposition, where low levels of sediment deposition (0.5 cm) may have buffered the adverse effects of warming through nutrient release and reduction of desiccation stress (Figure 4a) (Larson & Sundbäck, 2012; McKew et al., 2011; Nakamoto et al., 2000). The correlation effect evanished with increasing sediment deposition, weakening the interaction between sediment and warming. Increasing sediment loads may have generated hypoxic conditions at the sediment–biofilm interface, such that the antagonistic buffering effect caused by the 0.5 cm layer of sediment switched into a negative effect operating additively with warming under the thicker 1.5 cm layer. The correlation effect reversed under high warming and sediment deposition, with a positive spatial correlation of the two variables resulting in adverse effects on both the TVE and biofilm biomass compared with negative spatial correlation (Figures 4c and 5a,b). Such inversion of the correlation effect could also reflect a switch from an antagonistic to a synergistic effect of warming and sediment deposition on biofilm biomass. However, caution must be paid in interpreting this inversion, as no previous experiments have directly characterized the nature (antagonistic or synergistic) and the magnitude effect of warming and sediment deposition on rocky intertidal biofilm.

The observed differences among correlation scenarios might also reflect a shift in species composition. Although physiological responses to stress may occur within a few days in microorganisms, longer periods (months) may be necessary for these effects to translate into compositional shifts (Schimel et al., 2007). In a parallel experiment, we found that changes in species composition in response to warming required at least 4 months to be detected (L. Rindi, unpublished data). Since the experiment presented here lasted 3 months, we are more inclined to believe that outcomes were driven more by physiological responses than shifts in species composition within the biofilm.

Although the direct manipulation of warming and sediment reproduced the effects of intensity and spatial correlation observed in the simulations, RS predictions may be affected by error propagation (Figure 5). This likely reflected the uncertainty associated with estimating the RS from field data. However, results of the bootstrapping analysis showed that despite a moderate fit (*R* ^2^ = 0.29), our RS generated realistic predictions of response of biofilm to changes in intensity and spatial correlation of warming and sediment deposition.

Our study examined the effects of nonlinearities and correlation between variables at small spatial scales. Whether our RS could predict the TVE at larger spatial scales remains an open question. Scale‐transition theory uses Jensen's inequality (nonlinear averaging) and measures of environmental variances and correlations to extrapolate local ecological patterns (e.g., population dynamics within patches of habitat) to broader scales (e.g., regional population dynamics) (Benedetti‐Cecchi et al., 2012; Chesson et al., 2005; Melbourne & Chesson, 2006). The effect of Jensen's inequality increases with the degree of nonlinearities and with the amount of variance encountered when embracing larger spatial or temporal scales. Thermal performance curves are a typical example of nonlinear response functions that have been widely used to model population responses in fluctuating environments (Denny, 2017; Kingsolver & Woods, 2016; Koussoroplis & Wacker, 2016). Studies have shown how the shape of performance curves may change depending on the duration and history of exposure to stressful conditions, acclimatation, and ontogeny that may all affect the accuracy of temporal predictions from Jensen's inequality (Kingsolver & Woods, 2016; Kremer et al., 2018; Sinclair et al., 2016). Similar effects may occur when extrapolating across spatial scales. Due to the patchy nature of the rocky intertidal environment, biofilm assemblages might become increasingly different in terms of disturbance legacies, acclimatation, and overall response to warming and sediment deposition with increasing spatial scales, compromising the ability of the RS to predict patterns at larger scales. Although these caveats remain to be clarified, our study shows how consideration of nonlinear response functions and spatial correlation can help elucidating the influence of multiple processes at small spatial scales in a heterogeneous environment.

Focusing on local patterns and processes is important because small‐scale variability is ubiquitous in nature, and most of the interactions between organisms and the surrounding environment occur at the scale of the microhabitat (Korell et al., 2021; Pincebourde et al., 2016; Potter et al., 2013). Yet, microhabitat heterogeneity can promote adaptation by buffering populations against adverse environmental conditions and small‐scale processes can drive large‐scale patterns of community stability (Grman et al., 2010; Riddell et al., 2021). For example, ridges and depressions may generate various patterns of correlation among solar radiation, freezing, and moisture in tundra systems, providing favorable microclimates for the persistence, growth, and adaptation of dominant shrub species that are responsible for coarse‐scale vegetation shifts (greening) in Arctic and Alpine ecosystems (Dobbert et al., 2021). Similar small‐scale patterns of correlation between leading environmental variables are expected to occur in other terrestrial and aquatic systems where landscape features and topographic complexity promote fine‐scale environmental heterogeneity (Deák et al., 2021). Assessing the generality of nonlinear and correlation effects as mechanisms shaping small‐scale ecological patterns is important to better understand the compound effects of multiple processes and the ecological role of microclimates in changing environments.

Climate change projections for the 21st century involve modifications of the spatiotemporal patterns of climate variables (Gunderson et al., 2016; Hayashida et al., 2020; Young & Ribal, 2019). The spatial context in which organisms are embedded, such as landscape and microtopographic features, strongly filter and modify the climate‐change signal (Pincebourde et al., 2016). The thermal mosaics originating from the topographic complexity of rocky intertidal shores are an example of a filtered signal (Helmuth et al., 2006). In the same vein, filtered signals may originate in a multifactorial context when topographic features generate small‐scale patterns of correlation of environmental variables. As we have shown here, local patterns of correlation may play a crucial role in modulating the effect of climate extremes, ultimately influencing average biofilm biomass at the scale of the shore. Our analysis signals the need for researchers, resource managers, and policymakers aiming at predicting the impact of multiple stressors to account for current and possibly future spatiotemporal patterns of correlation among stressors. Black swans can occur in space as well as in time (Anderson & Ward, 2019), but the spatial context is often overlooked in the analysis of ecological extremes. Spatial correlation should be explicitly incorporated into multiple‐stressors studies (Gunderson et al., 2016), risk‐assessment framework (Côté et al., 2016; Goussen et al., 2016) and coupled environmental‐physiological models (Pincebourde et al., 2016; Rezende et al., 2014). Our study shows how the inclusion of correlation between drivers can improve predictions from Jensen's inequality in real‐world conditions. Further experimental work is needed to evaluate the generality of these findings in other ecosystems and over a wider range of environmental variables.

## AUTHOR CONTRIBUTIONS

**Jianyu He:** Data curation (equal); methodology (equal). **Lisandro Benedetti‐Cecchi:** Conceptualization (equal); funding acquisition (lead); project administration (lead); resources (lead); supervision (equal); writing – original draft (equal); writing – review and editing (equal).

## CONFLICT OF INTEREST

Authors declare no conflict of interest.

## Supporting information


Appendix S1

## Data Availability

Data are available on Figshare at the following https://doi.org/10.6084/m9.figshare.14447871.
