# Supplementary material for: Spatial correlation reverses the compound effect of multiple stressors on rocky shore biofilm
Source: Ecol Evol. 2022 Oct 27;12(10):e9418. doi: 10.1002/ece3.9418 (PMC9608791; doi:10.1002/ece3.9418)
Supplement: Supplementary file 1 — Appendix S1 [file ECE3-12-e9418-s001.docx]

**Supporting Information**

**Spatial correlation reverses the compound effect of multiple stressors on rocky shore biofilm**

L. Rindi^1^, J. He^1^, L. Benedetti-Cecchi^1^

^1^Department of Biology, University of Pisa, CoNISMa, Via Derna 1, Pisa, Italy

**Appendix S1**

**Supplemental Materials and Methods**

**Estimation of the return-time of experimental warming levels**

Positive temperature anomalies with respect to the 1951-1980 reference period were determined using 66 yrs (1951-2017) timeseries of air temperature collected by the Consorzio LaMMA Toscana (http://www.lamma.rete.toscana.it) and Rete Mareografica Nazionale (ISPRA - Istituto Superiore per la Ricerca e la Protezione Ambientale). Return time of positive temperature anomalies were estimated along with their confidence intervals by fitting a Generalized Extreme Values (GEV) distribution to time series of daily maximum air temperatures for the period corresponding to the months in which the heating treatments were imposed (March to August) (Katz, Brush, and Parlange 2005). This analysis defined the treatment warming levels (+5, +10 and +15 °C) used in the response-surface design (Figure S3).

**Supplemental references**

Katz, Richard W., Grace S. Brush, and Marc B. Parlange. 2005. “Statistics of Extremes: Modeling Ecological Disturbances.” *Ecology* 86 (5): 1124–34. https://doi.org/10.1890/04-0606.

Wood, Simon N. 2013. “On P-Values for Smooth Components of an Extended Generalized Additive Model.” *Biometrika* 100 (1): 221–28. https://doi.org/10.1093/biomet/ass048.

**Supplementary Figures**

**
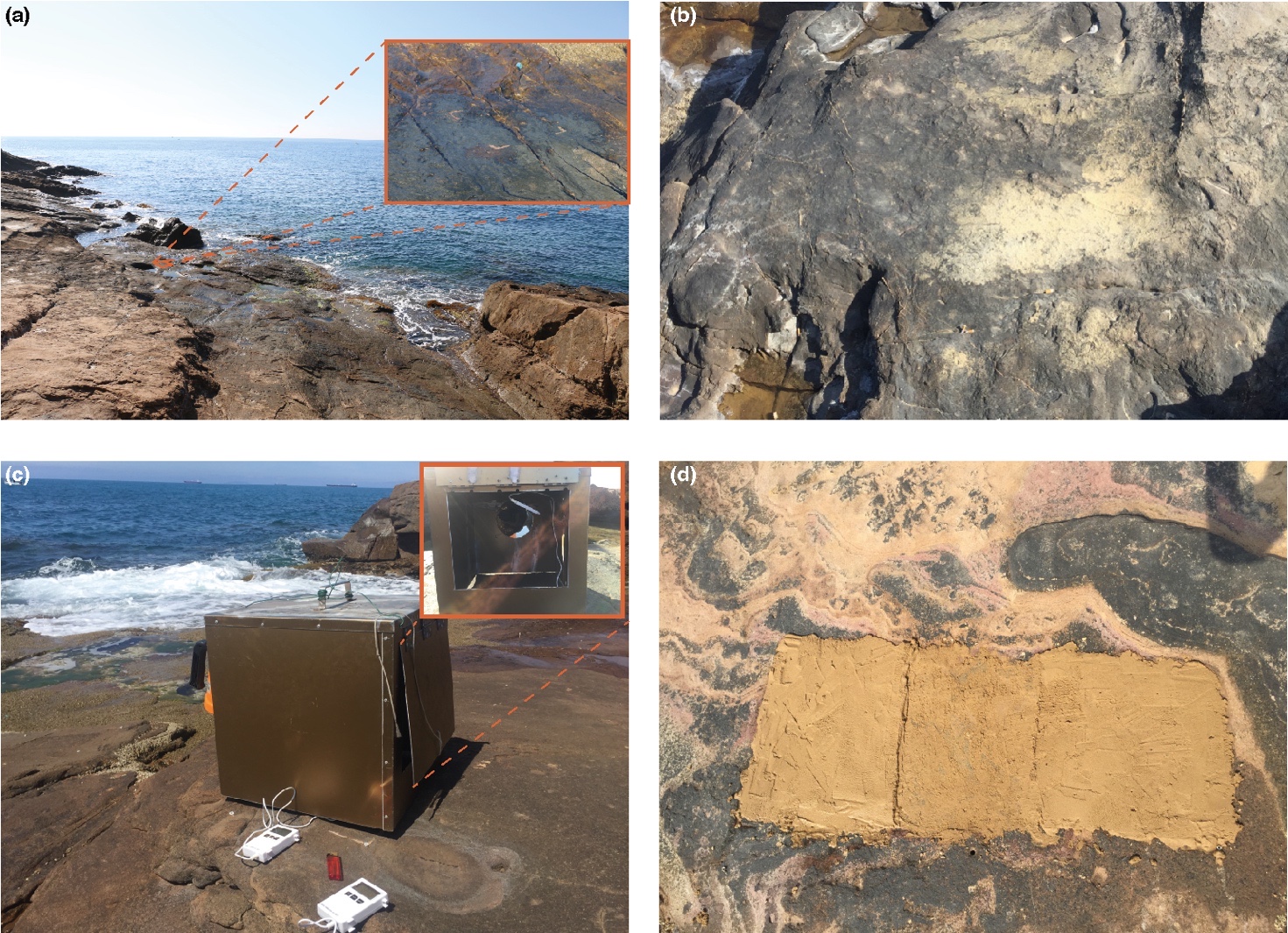
**

**Figure S1**. **Study site and experimental treatments.** (a) Landscape view of the study site (June 2017). The biofilm is visible as dark areas on the rock. The inset represents a close-up of an experimental unit (plot of 40 × 40 cm). (b) Sediment accretion following a heavy rainfall event. The micro-topography of the substratum generates a mosaic of areas characterized by different levels of sediment accumulation and temperature resulting in positive or negative patterns of spatial correlation between these drivers of biofilm biomass. (c) Aluminium chamber equipped a with low-power butane heater. The close-up shows the data loggers (button FT 800/System ©) used to record air temperature inside the chambers, together with a digital thermometer that was used to monitor air temperatures outside the chambers during the 2 h warming session. (d) An experimental transect treated with a thick layer of sediment (1.5 cm on average) surrounded by a thin layer of natural sediment.

**
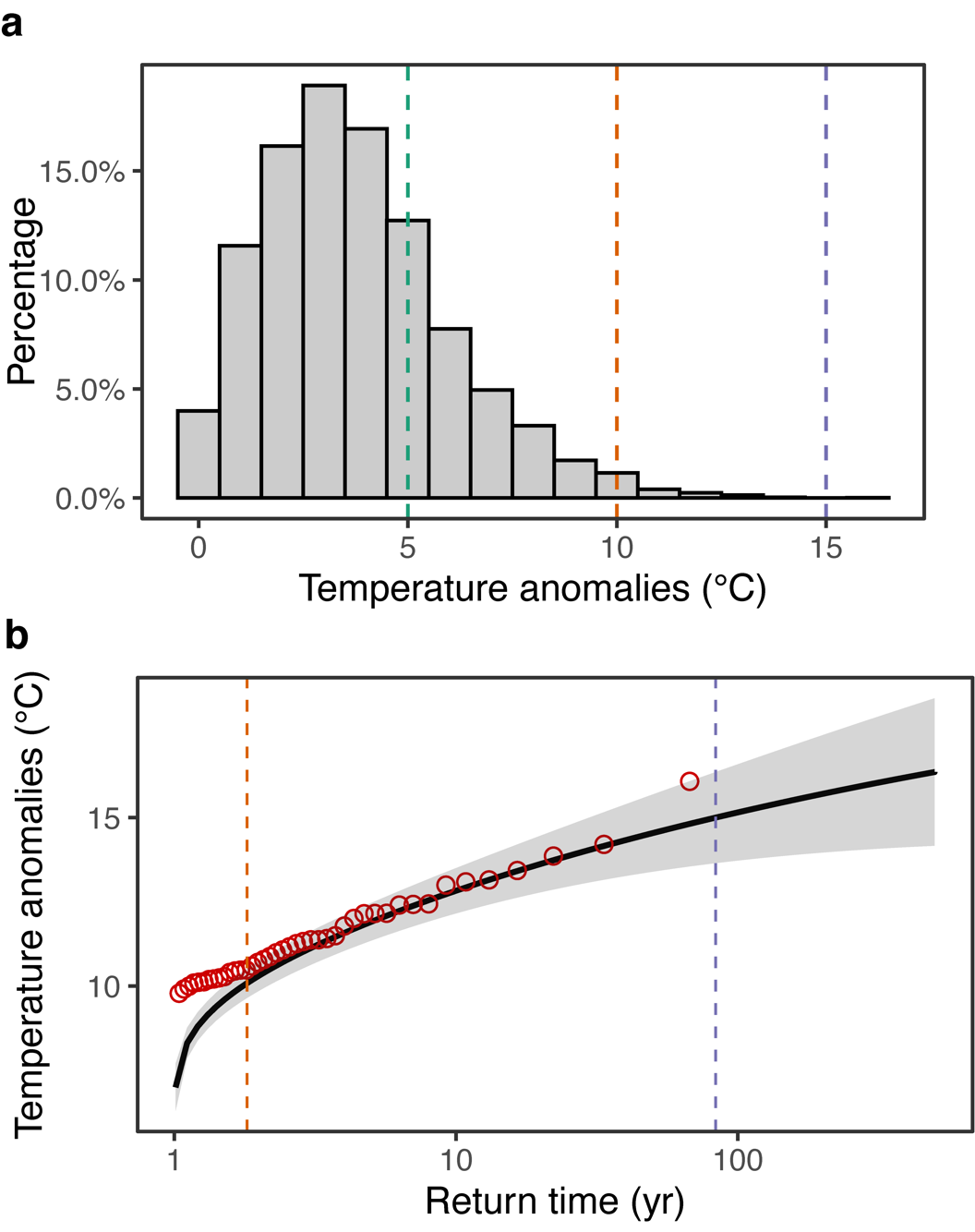
**

**Figure S2**. **Temperature anomalies and their return time.** (a) Frequency distribution of positive temperature anomalies calculated using a 66-yr timeseries (1951-2017). Dashed lines indicated the three levels of warming used in the response-surface experiment. (b) Temperature anomalies as a function of return time (in years). The red and blue dashed lines indicate the return times of +10 °C and +15 °C of warming, respectively. The lowest warming treatment (+5 °C) is not shown, as it has a return time shorter that one year.


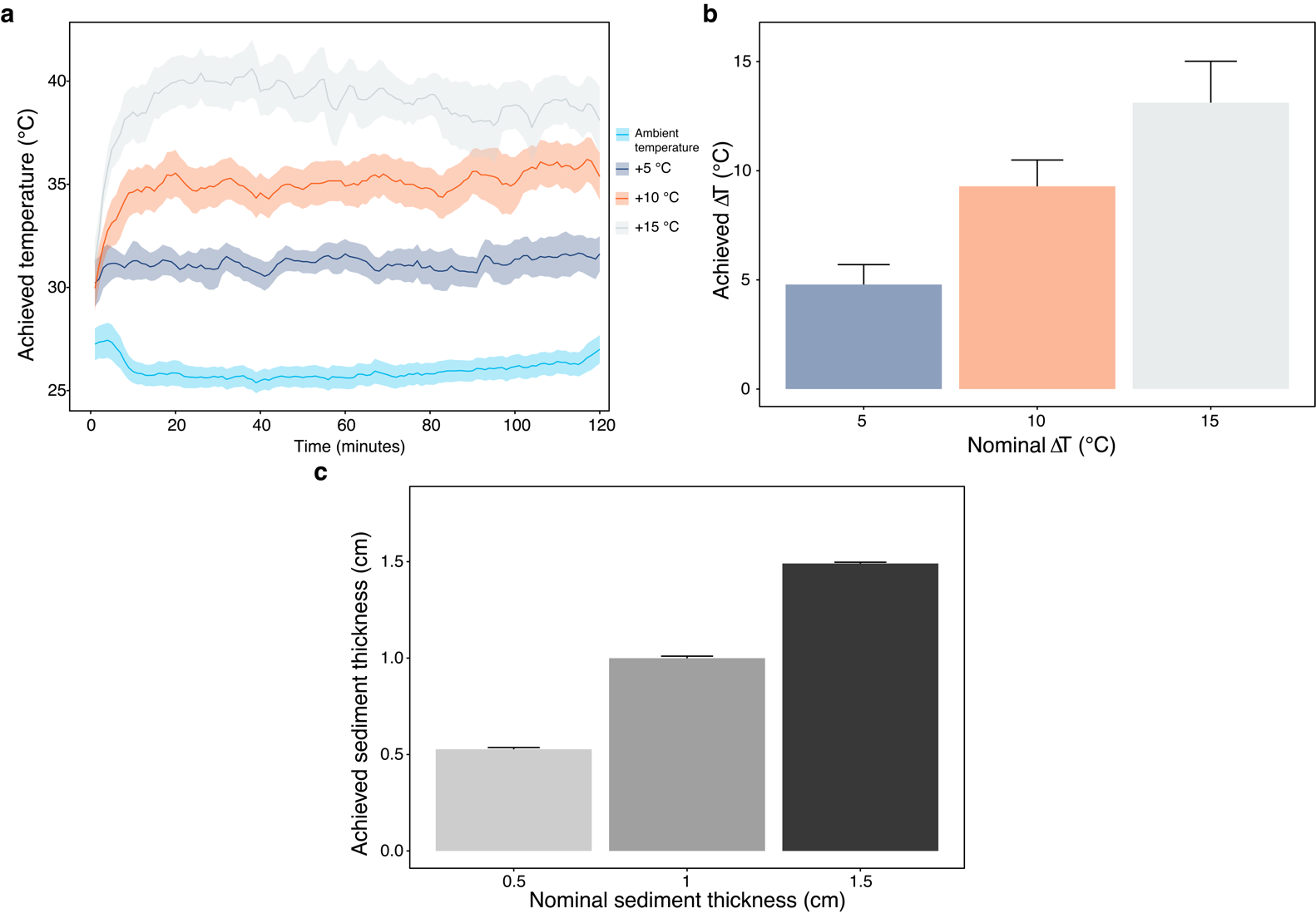


**Figure S3**. **Experiment 1:** **realized vs. nominal treatment levels.** (a) Temporal profiles of mean air temperature (averaged over three replicate plots and four levels of sediment deposition) for four warming conditions: control (ambient temperature; cyan), +5 °C (blue), +10 °C (red) and +15 °C (grey) above ambient temperature. Shaded regions correspond to ± 1SE (*n*=12). (b) Achieved *vs* nominal levels of warming (ΔT above ambient temperature; means +1SE, *n*=12). (c) Achieved *vs.* nominal levels of sediment thickness (means +1SE, *n*=12).

**
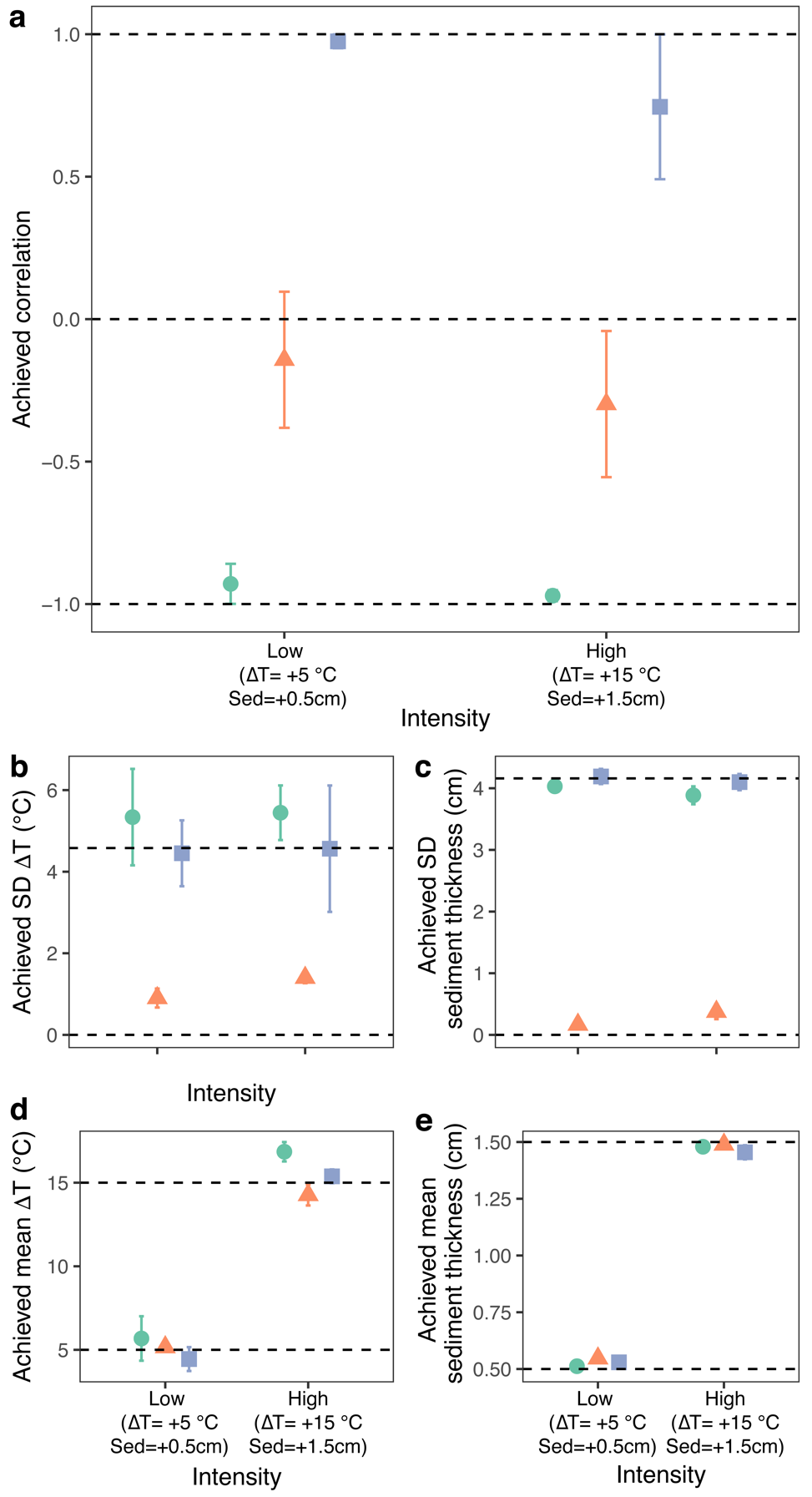
**

**Figure S4**.**Testing predictions: achieved *vs*. nominal treatment levels.**Low: 30.4 °C warming**(**ΔT = 5 °C above ambient temperature), sediment thickness= +0.5 cm; High: 40.0 °C warming**(**ΔT = 15°C above ambient temperature), sediment thickness= +1.5 cm. (a) Correlation between warming and sediment deposition. (b) Standard deviations of warming (ΔT) and (c) thickness of the sediment layer (d) Realized means of warming (ΔT) and (e) thickness of the sediment layer. Dashed lines in different panels indicate nominal values. Error bars are ±1SE (*n*=3).


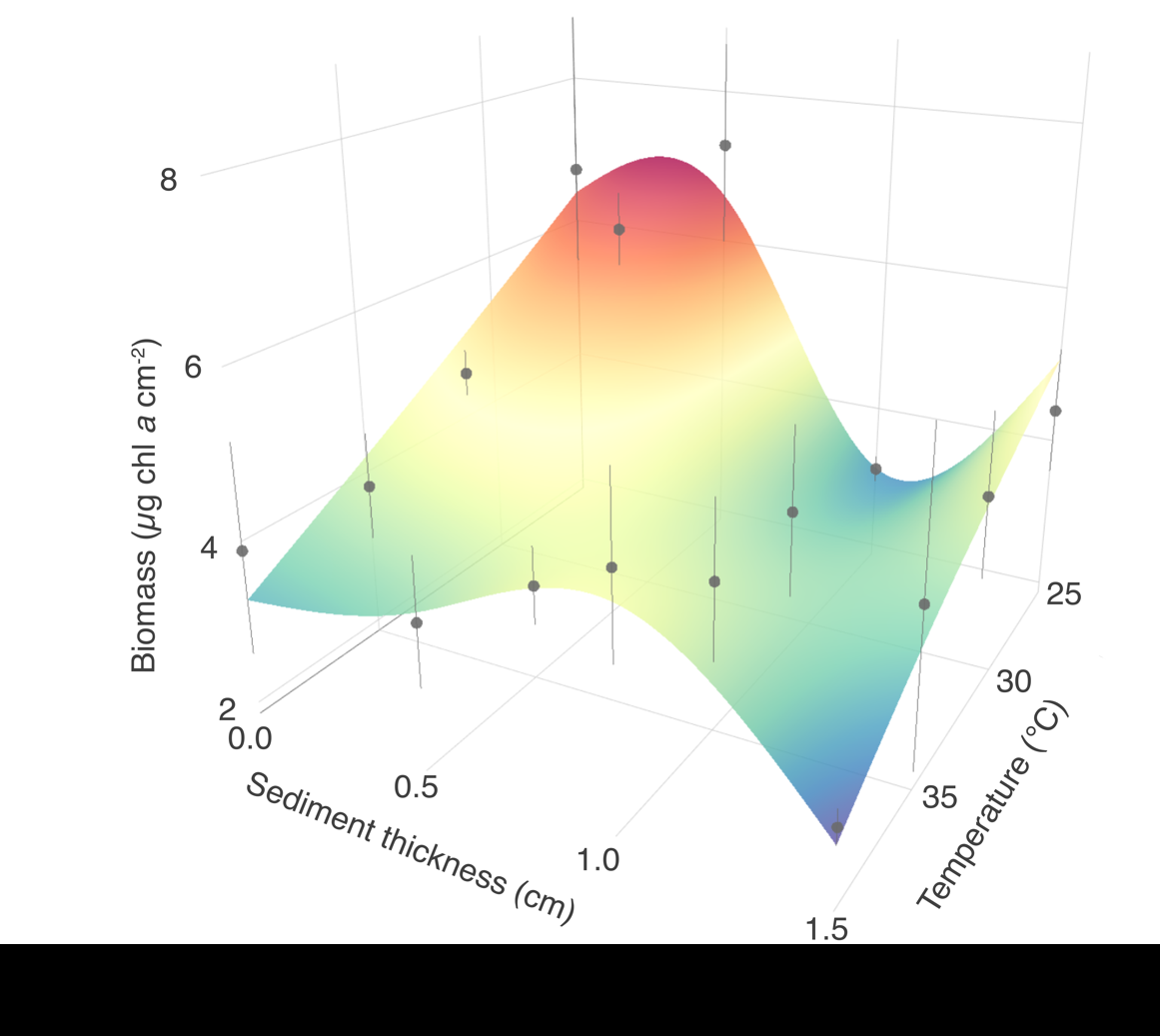


**Figure S5. Chlorophyll *a* as a function of absolute temperature (T) and sediment deposition.** Temperature-sediment response surface (RS). Mean + SE (*n*=3) biofilm biomass (µg chl *a* cm^-2^) for each warming-sediment combination. The response surface derived from absolute temperatures has a poorer fit than the one fitted to delta temperatures, defined as the degree of experimental warming above ambient temperature (absolute temperature: AIC=178.71, R^2^_Adj._ = 27.7%; delta temperature: AIC=175.0, R^2^_Adj._ = 29%) (Tab S1, Fig. 2). Despite the slight difference in the goodness of fit, the two RS show very similar nonlinear patterns.


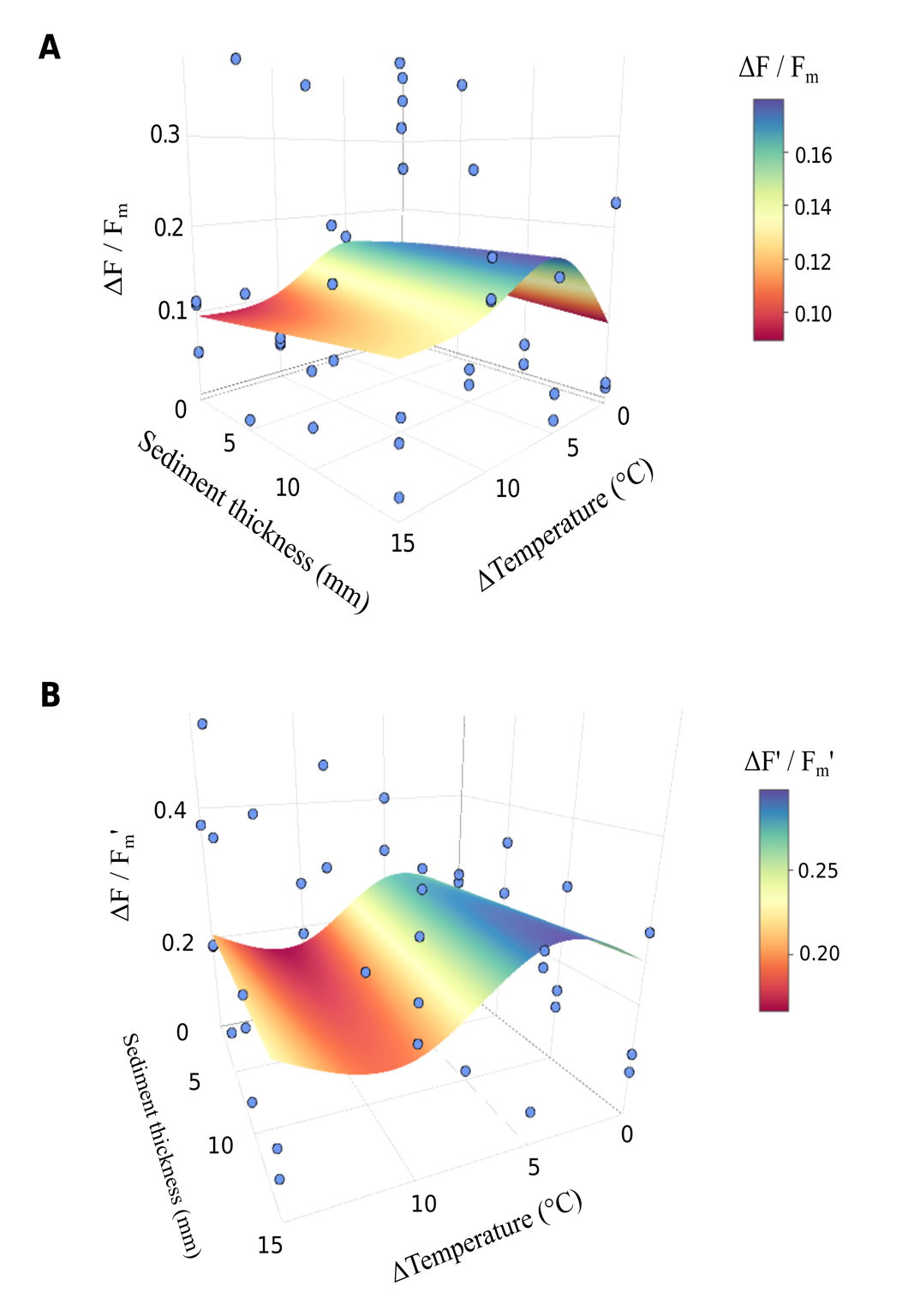


**Figure S6. Photosynthetic efficiency.** Panels show the response surfaces obtained after dark adaption (a) and under actinic light (b) as a function of warming (∆T: °C above ambient temperature) and sediment deposition. Blue points represent the mean of each experimental plot over the three sampling dates (*n*=3).

**
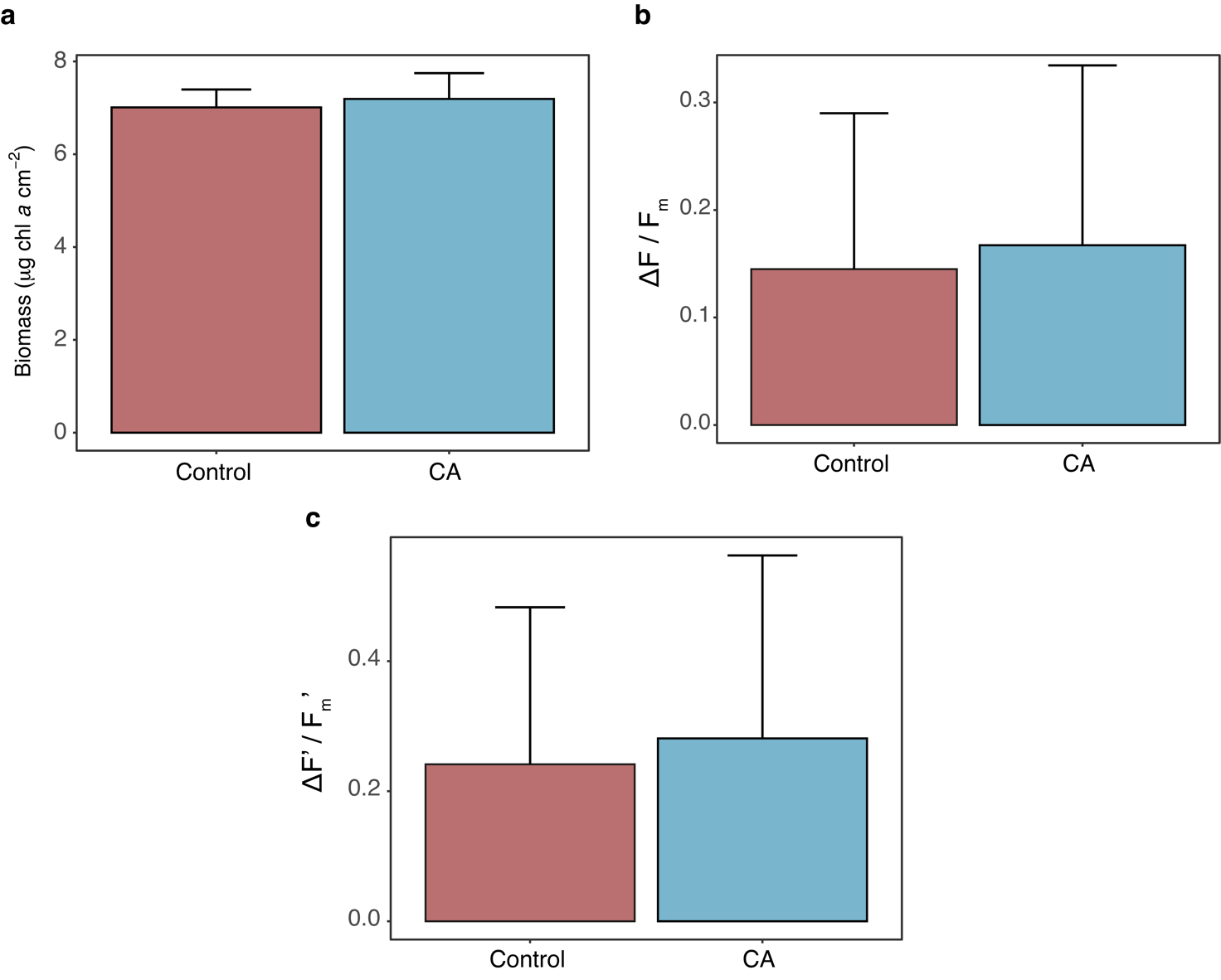
**

**Figure S7. Evaluation of experimental artefacts in the first experiment.** The panels show the mean values of biofilm biomass (a), dark yield ($\Delta F/F_{m}$) (a) and light yield ($\Delta F^{'}/F_{m}^{'}$) in controls and control for artefacts (CA) conditions. CA plots were shaded with cardboard chambers, but they were not warmed. Mean + SE (*n*=3).


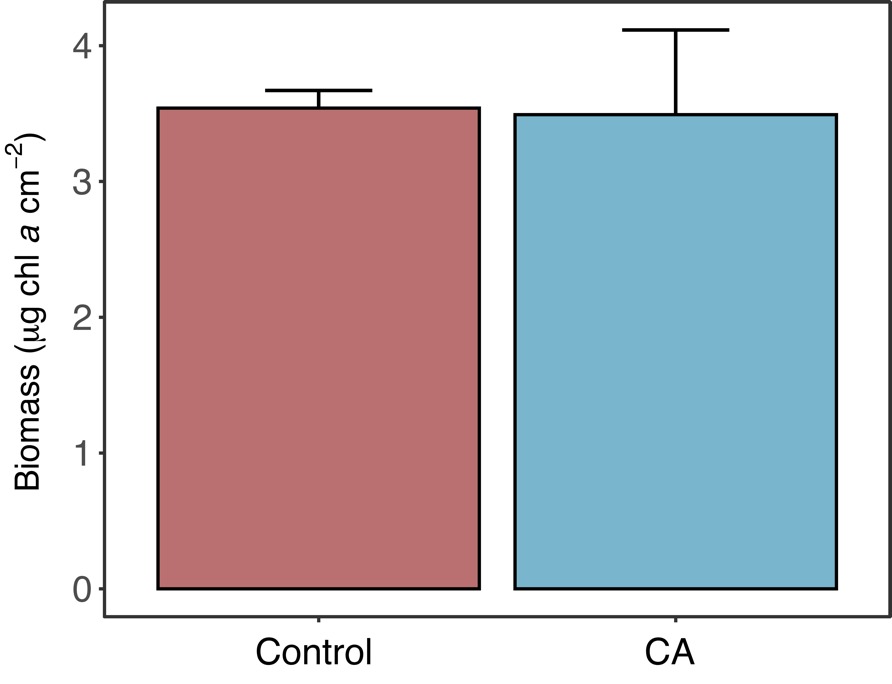


**Figure S8. Evaluation of experimental artefacts in the second experiment.** The figure shows the mean values of biofilm biomass in controls and control for artefacts (CA) conditions. CA transects were shaded with cardboard chambers, but they were not warmed. Mean + SE (*n*=3).

**Supplementary Tables**

**Table S1. General Additive Model (GAM) of biofilm biomass (µg chl *a* cm^-2^).** “s”: function used for the definition of smooth terms within the GAM formula; “te” function used for the definition of tensor product smooth and interaction terms within the GAM formula; edf: effective degrees of freedom; GCV: minimized generalized cross validation score for the model; Scale est.: estimated scale parameter. Significant differences are reported in bold. Significance of model term is assessed using a Wald like test, conditional on smoothing parameters estimates (Wood 2013).

|  | **edf** | ***Wald*** | ***p*** |
| --- | --- | --- | --- |
| Warming = Warm | 1.00 | 5.85 | **<0.01** |
| Sediment deposition = Sed | 2.97 | 5.12 | **<0.01** |
| Warm x Sed | 1.85 | 1.09 | **<0.01** |
| *R^2^* | 0.29 | | |
| GCV | 2.73 | | |
| Deviance explained | 38.20 % | | |
| Scale est. | 2.33 | | |

**Table S2. General Additive Model (GAM) dark yield (∆F/F_m_) and light yield (∆F'/F_m_').** “s”: function used for the definition of smooth terms within the GAM formula; “te” function used for the definition of tensor product smooth and interaction terms within the GAM formula; edf: effective degrees of freedom; GCV: minimized generalized cross validation score for the model; Scale est.: estimated scale parameter. Significant differences are reported in bold. Significance of model term is assessed using a Wald test statistic.

| **Maximum photochemical efficiency after dark adaption**  **(*∆F*/*F_m_*)** |  | **edf** | ***Wald*** | ***p*** |
| --- | --- | --- | --- | --- |
|  | Warming = Warm | 1.00 | 0.44 | >0.40 |
|  | Sediment deposition = Sed | 2.50 | 1.48 | >0.30 |
|  | Warm x Sed | 0.50 | 0.09 | >0.15 |
|  | *R^2^* | 0.042 | | |
|  | GCV | 0.014 | | |
|  | Deviance explained | 12.3 % | | |
|  | Scale est. | 0.0125 | | |
| **Effective quantum yield of photosystem II in actinic light**  **(*∆F'*/*F_m_'*)** | Warming = Warm | 1.00 | 0.34 | >0.50 |
|  | Sediment deposition = Sed | 2.77 | 2.06 | >0.10 |
|  | Warm x Sed | 2.60 | 0.00 | >0.90 |
|  | R^2^ | 0.0744 | | |
|  | GCV | 0.0123 | | |
|  | Deviance explained | 14.9 % | | |
|  | Scale est. | 0.011 | | |

**Table S3**. Results of Linear Mixed Effect Models (LMEM) used to assess the effects of intensity of warming and sediment deposition and their spatial correlation on biofilm biomass and on the Total Variance Effect (TVE). Correlation: Cor (two levels: Negative and Positive) and Intensity: Int Cor (two levels: Low and High). Post-hoc contrasts performed with the R function *emmeans* are included for significant “Cor x Int” interaction.

|  |  | | | | | **Estimate (SE)** | | | **Df** | **t-value** | | | | | ***p*** |  |
| --- | --- | --- | --- | --- | --- | --- | --- | --- | --- | --- | --- | --- | --- | --- | --- | --- |
| **Biomass**  **(Chl *a* µg cm^-2^)** | Intercept (Cor. N - Int. L) | | | | | 1.48 (0.21) | | | 12 | 6.97 | | | | | **<0.001** | |
|  | Cor. P | | | | | -0.86 (0.30) | | | 12 | -2.87 | | | | | **<0.05** | |
|  | Int. L | | | | | -0.86 (0.30) | | | 12 | -1.38 | | | | | >0.15 | |
|  | Cor. P x Int. L | | | | | 1.27 (0.43) | | | 12 | 2.98 | | | | | **<0.05** | |
|  |  | | | | |  | | |  |  | | | | |  | |
|  | **Random Effects** | | | | | **Std. Dev.** | | |  | | | | | |  |  |
|  | Transect | | | | | 0.40 | | |  | | | | | |  |  |
|  | Residuals | | | | | 0.46 | | |  | | | | | |  |  |
|  |  | | | | |  | | |  | | | | | |  |  |
|  | ***Post-hoc contrasts*** | | | | |  | | | | | | | |  | |  |
|  | Low intensity | | | | **Estimate (SE)** | | **Df** | ***t-ratio*** | | | ***p*** | |  | | |  |
|  | Cor-1*vs* Cor+1 | | | | -0.40 (0.37) | | 18 | -1.10 | | | >0.20 | |  | | |  |
|  | High intensity | | | |  | |  |  | | |  | |  | | |  |
|  | Cor-1*vs* Cor+1 | | | | 0.86 (0.37) | | 18 | 2.34 | | | **<0.05** | |  | | |  |
|  |  |  |  |  |  | | | | | | |  |  |  |  |  |
|  |  |  |  |  | Transformation log(x+0.1) | | | | | | |  |  |  |  |  |

|  |  | | | | | **Estimate (SE)** | | | **Df** | **t-value** | | | | | ***p*** |  |
| --- | --- | --- | --- | --- | --- | --- | --- | --- | --- | --- | --- | --- | --- | --- | --- | --- |
| **Total Variance Effect (TVE)** | Intercept (Cor. N - Int. L) | | | | | 5.04 (0.54) | | | 12 | 9.34 | | | | | **<0.001** | |
|  | Cor. P | | | | | -2.17 (0.76) | | | 12 | -2.85 | | | | | **<0.05** | |
|  | Int. L | | | | | -2.65 (0.76) | | | 12 | -3.47 | | | | | **<0.01** | |
|  | Cor. P x Int. L | | | | | 3.49 (1.08) | | | 12 | 3.23 | | | | | **<0.01** | |
|  |  | | | | |  | | |  |  | | | | |  | |
|  | **Random Effects** | | | | | **Std. Dev.** | | |  | | | | | |  |  |
|  | Transect | | | | | 0.83 | | |  | | | | | |  |  |
|  | Residuals | | | | | 0.74 | | |  | | | | | |  |  |
|  |  | | | | |  | | |  | | | | | |  |  |
|  | ***Post-hoc contrasts*** | | | | |  | | | | | | | |  | |  |
|  | Low intensity | | | | **Estimate (SE)** | | **Df** | ***t-ratio*** | | | ***p*** | |  | | |  |
|  | Cor-1*vs* Cor+1 | | | | -1.31 (0.93) | | 18 | -1.33 | | | >0.15 | |  | | |  |
|  | High intensity | | | |  | |  |  | | |  | |  | | |  |
|  | Cor-1*vs* Cor+1 | | | | 2.17 (0.93) | | 18 | 2.32 | | | **<0.05** | |  | | |  |
|  |  |  |  |  |  | | | | | | |  |  |  |  |  |
|  |  |  |  |  | Transformation log(x+\|min(x)\|+1) | | | | | | |  |  |  |  |  |

**Table S4**. *t*-tests assessing shading effects of the aluminium chambers in the first experiment.

|  |  | | **Df** | | ***t-value*** | ***p*** |  |
| --- | --- | --- | --- | --- | --- | --- | --- |
| **Biomass (Chl *a* µg cm^-2^)** | | 3.49 | | -0.01 | | >0.90 | |
|  | | 3.99 | | -0.54 | | >0.60 | |
|  | | 2.05 | | -0.50 | | >0.60 | |

**Table S5**. Results of Linear Mixed Effect Models (LMEM) used to evaluate shading effects of the aluminium chambers in the second experiment.

|  |  | | **Estimate (SE)** | | **Df** | | ***t-value*** | | | | ***p*** |  |  |
| --- | --- | --- | --- | --- | --- | --- | --- | --- | --- | --- | --- | --- | --- |
| Intercept (Control) | | 1.27 (0.05) | | 12 | | 23.00 | | | **<0.001** | | | |  |
| Control *vs* CA | | -0.03 (0.11) | | 12 | | | | -0.32 | | >0.70 | | |  |
|  | |  | |  | | | |  | |  | | |  |
| **Random Effects** | | **Std. Dev.** | |  | | | |  | |  | | |  |
| Transect | | 0.00 | |  | | | |  | |  | | |  |
| Residuals | | 1.63 | |  | | | |  | |  | | |  |
